# High awareness of diabetes in the health care system in Greenland measured as a proportion of population tested with glycated haemoglobin within 2 years

**DOI:** 10.1186/s13098-017-0230-4

**Published:** 2017-05-03

**Authors:** Michael Lynge Pedersen

**Affiliations:** 1 Greenland Center of Health Research, Institute of Nursing and Health Science, University of Greenland, Nuuk, Greenland; 2 Queen Ingrid Primary Health Care Center, Box 3333, 3900 Nuuk, Greenland

**Keywords:** Diabetes, Glycated haemoglobin, Diagnosis, Prevalence

## Abstract

**Background:**

Sixty years ago diabetes was almost non-existent in Greenland and until the beginning of this century awareness of diabetes was quite minimal. A high prevalence of undiagnosed diabetes has been reported in repeated population surveys. Increased focus on diabetes has been made a priority within the health care system since 2008, and in 2010 glycated haemoglobin was introduced as a diagnostic tool to further facilitate the diagnosis of diabetes.

**Objective:**

The aim of this study was to estimate the age and gender specific use of glycated haemoglobin in 2014 and 2015, as an indicator of diagnostic activity and awareness of diabetes, and to estimate the prevalence of diagnosed pre-diabetes and diabetes among adults in Greenland aged 20–79 years of age.

**Methods:**

The study was performed as an observational, cross sectional register study based on information gleaned from the electronically laboratory system used in Greenland including all patients tested with glycated haemoglobin at least once in 2014 or 2015.

**Results:**

A total of 10,127 patients were tested with glycated haemoglobin in 2014 or 2015 corresponding to 18.1% of the whole population. Among adults aged 20–79 years 9506 patients were tested corresponding to 24.0% of the total adult population. More females (32.5%) than males (16.5%) aged 20–79 years old were tested (p < 0.001). The prevalence of diagnosed diabetes and high risk pre-diabetes among adults aged 20–79 years was 4.3 and 6.8% respectively.

**Conclusion:**

In conclusion use of glycated haemoglobin is widely used in the health care system in Greenland indicating a high awareness of diabetes in the population and by the health care system. Still, awareness of undiagnosed diabetes remains an important issue and additional strategies targeting males under 70 years old must be considered.

## Background

Diabetes is a serious and increasing health challenge affecting in 2013 around 382 million people globally [1]. The number is projected to increase with approximately 55% to 592 million within the next 20 years [1, 2]. Nine percent of adults at or above 18 years old are affected by diabetes [3]. Due to the often slow onset of symptoms for especially type 2 diabetes the condition may go undiagnosed for several years. Worldwide almost half of all diabetes cases (175 million people) remain undetected [2]. During the undiagnosed period, elevated blood glucose may lead to microvascular complications like neuropathy, nephropathy, retinopathy and macrovascular complications like ischemic heart disease, stroke and peripheral vascular disease [2, 4, 5]. The majority of both diagnosed and undiagnosed cases of diabetes are in low and middle income countries [1, 2].

Particularly rapid development of diabetes has been reported worldwide among Indigenous peoples [6]. Also in Greenland the prevalence of diabetes has been increasing [7, 8]. Sixty years ago diabetes in Greenland was almost non-existent [9, 10], and not much attention to diabetes was observed until this century. However, a population health survey including 917 participants performed around 2000 in Greenland indicated a high prevalence of diabetes based on oral glucose tolerance tests from among Greenlanders [11, 12], levels comparable to those levels found among Inuit and Native Indian populations in Canada and Alaska [13]. Thus, around 10% of adult at or above 35 years old were reported to suffer from diabetes and an additional 20% (approximate) from impaired glucose tolerance. Furthermore, more than 70% of the diabetes cases reported in the population survey were undiagnosed [11]. These findings led to an increased focus on diabetes in Greenland in the health care system in Greenland [13–15]. The healthcare system in Greenland is divided into five healthcare regions. Primary health care is delivered by a health care clinic in each town in co-operation with small health care units located in the adjacent settlements. Queen Ingrid Hospital is located in the capital of Nuuk and functions as the central hospital for all Greenland, and delivers secondary specialized health care. Health care is free to anyone with permanent residence in Greenland. Diabetes health care has been reported to be improved and the prevalence of diagnosed diabetes has been increasing since 2008 [8]. In 2010, HbA1c was introduced as a screening tool in Greenland in order to optimize diagnostic activity [7]. All pregnant women in Greenland are routinely tested with HbA1c at first trimester. No other routine screening is performed. However, patients diagnosed with diabetes, pre-diabetes, hypertension and cardio-vascular diseases, obesity and other chronic conditions are comprehensively offered to be tested with HbA1 by health care providers. However, the actual use of HbA1c 5 years after the introduction has not been evaluated and diagnostic activity in Greenland remains unknown. Thus, the aim of this study was to estimate the age and gender specific use of glycated haemoglobin in 2014 and 2015 as an indicator of diagnostic activity and awareness of diabetes, and to estimate the prevalence of diagnosed pre-diabetes and diabetes among adults aged 20–79 years of age in Greenland.

## Methods

The study was performed as an observational, cross sectional register study based on statistic information taken from the electronically laboratory systems used in Greenland. Approximately 56,000 people live in Greenland, and the population is widely spread geographically along the coast in 17 towns and 60 settlements. Around 90% of the current population was born in Greenland [17].

A statistical extraction identifying all patients that have been tested in Greenland with an HbA1c test within 2014 and 2015 was performed. Only persons aged 20–79 years of age were included, while younger and older persons were excluded. Only the most recent value of HbA1c and only tests performed at the central laboratory at Queen Ingrid Hospital in Nuuk were included in the study. HbA1c level was measured through analysis of venous blood, performed at the central laboratory at Queen Ingrid Hospital in Nuuk, using Tosoh G8 HPLC analyser [16]. The Central Laboratory is a member of the Danish Quality Control System for laboratories [8]. Patients with an elevated value of HbA1c at or above 6.5% (48 mmol/mol) were considered having diabetes while patients with HbA1c from 5.7 to 6.4% (39–47 mmol/mol) were considered having pre-diabetes [18]. Finally, patients with a HbA1c value from 6.1 to 6.4% (42–47 mmol/mol) were considered having high risk pre-diabetes [18]. HbA1c values below 5.7% (42 mmol/mol) were considered normal. The age and gender specific prevalence of patients tested with HbA1c and the prevalence of diagnosed diabetes and pre-diabetes was calculated using the background population in Greenland as of first of January 2015.

Estimates were calculated with 95% confidence intervals (CI). A z-score for two population proportions was calculated to compare for different age and gender groups. A p-value below 0.05 was used as the level of significance.

## Results

A total of 10,127 patients (6346 females and 3781 males) who had been tested with HbA1c at Queen Ingrid Hospital at least once in 2014 or 2015 were identified, corresponding to 18.1% of the whole population. Among adults aged 20–79 years, 9506 patients (3570 males and 5936 females) were tested. This corresponds to 24.0% of the total adult population. Of those, 52.6% (1885 of 3570 tested males and 3074 of 5936 tested females) with no gender difference observed (p = 0.337) had pre-diabetes. The age and gender specific proportion of the total population tested with HbA1c, and the estimated prevalence of diabetes and high risk pre-diabetes are shown in Table 1. Females were tested more often than males in all age groups below 70 years old (see Table 1). The prevalence of diagnosed diabetes and high risk pre-diabetes among all adults aged 20–79 years was 4.3 and 6.8%. The prevalence of diagnosed diabetes and high risk pre-diabetes was higher among all females than among all males aged 20–79 years (see Table 1). Both the prevalence of high risk pre-diabetes and diabetes was seen rising with age.

**Table 1:** Age and gender specific proportion of total population in Greenland tested with HbA1c during 2014 or 2015 and estimated prevalence of diagnosed diabetes and pre-diabetes

Age, years	HbA1c test in 2014–2015 % (95 % CI) (n/N)				High risk pre-diabetes % (95 % CI) (n)				Diabetes % (95 % CI) (n)			
	Total	Males	Females	p (Z-score)	Total	Males	Females	p (Z-score)	Total	Males	Females	p (Z-score)
20–39	16.6	5.5	28.4	*<0.001*	2.5	1.2	3.9	*<0.001*	0.5	0.4	0.6	0.097
	(16.6–17.2)	(5.1–5.9)	(27.4–29.4)	(−38.9)	(2.3–2.8)	(1.0–1.5)	(3.5–4.4)	(−11.0)	(0.4–0.6)	(0.2–0.59	(0.4–0.7)	(−1.7)
	(2671/16,060)	(459/8272)	(2212/7788)		(408)	(101)	(307)		(74)	(31)	(43)	
40–49	18.4	12.0	26.0	*<0.001*	5.5	3.6	7.7	*<0.001*	2.1	1.9	2.4	0.159
	(17.5–19.2)	(11.0–12.9)	(24.6–27.4)	(−16.4)	(5.0–6.0)	(3.1–4.2)	(6.9–8.6)	(−8.1)	(1.8–2.4)	(1.5–2.3)	(1.9–2.9)	(−1.4)
	(1516/8251)	(538/4488)	(978/3763)		(452)	(162)	(290)		(175)	(86)	(89)	
50–59	28.6	22.7	36.0	*<0.001*	10.4	7.6	13.8	*<0.001*	6.4	5.5	7.6	*<0.001*
	(27.7–29.6)	(21.5–23.9)	(34.5–37.5)	(−13.8)	(9.7–11.0)	(6.9–8.4)	(12.7–14.8)	(−9.4)	(5.9–6.9)	(4.8–6.1)	(6.8–8.4)	(−4.0)
	(2523/8807)	(1103/4864)	(1420/3943)		(913)	(370)	(543)		(556)	(267)	(299)	
60–69	40.0	36.3	45.1	*<0.001*	13.6	10.8	17.4	*<0.001*	12.3	11.9	12.8	0.359
	(38.6–41.4)	(34.5–38.2)	(42.8–47.4)	(25.4)	(12.6–14.6)	(9.6–12.0)	(15.7–19.1)	(−6.3)	(11.3–13.3)	(10.7–13.2)	(11.3–14.4)	(−0.9)
	(1784/4460)	(941/2591)	(843/1869)		(605)	(280)	(325)		(549)	(309)	(240)	
70–79	50.1	51.0	49.2	0.412	15.3	14.2	16.5	0.148	16.5	17.1	15.9	*0.003*
	(47.9–52.3)	(48.0–54.1)	(46.1–52.3)	(0.8)	(13.7–16.9)	(12.1–16.3)	(14.2–18.8)	(−1.4)	(14.9–18.1)	(14.8–19.4)	(13.6–18.2)	(−3.0)
	(1012/2019)	(529/1037)	(483/982)		(309)	(147)	(162)		(333)	(117)	(156)	
Total (20–79)	24.0	16.8	32.4	*<0.001*	6.8	5.0	8.9	*<0.001*	4.3	4.1	4.5	*0.042*
	(23.6–24.4)	(16.3–17.3)	(31.7–33.0)	(−36.1)	(6.5–7.0)	(4.7–5.3)	(8.5–9.3)	(−15.3)	(4.1–4.5)	(3.8–4.4)	(4.2–4.8)	(−2.0)
	(9506/39,597)	(3570/21,252)	(5936/18,345)		(2824)	(1060)	(1627)		(1687)	(870)	(827)	

p-values below 0.05 (P < 0.05) are in Italic

## Discussion

Almost a fourth (24.0%) of the total adult population (20–79 years old) and almost a third (32.4%) of the adult females in Greenland had a HbA1c test performed within a 2 year observational period indicating high diagnostic activity in the health care system. More than half of all tested adult patients had pre-diabetes. The proportion of the population tested was higher for females than males. Accordingly, the prevalence of diagnosed diabetes and of high risk diabetes was higher among females than males (20–79 years old).

### Strengths and limitations

The main strength of the present study is that it is the first study to describe diagnostic activity in all of Greenland. Also, the number of patients included in this study is larger than any study ever published before in Greenland on the prevalence of high risk pre-diabetes and diabetes. However, several limitations exist. Some towns outside Nuuk perform HbA1c test on patients with diabetes locally instead of using the central laboratory of Queen Ingrid Hospital. These test results were not included in this study. Thus, the number of patients tested and the number of patients with diabetes may have been underestimated in this study. However, most patients with diabetes experience at least once annually additional blood tests including HbA1c, blood lipids and electrolytes tested and performed at the central laboratory of Queen Ingrid Hospital. Thus, the size of the underestimations can be regarded as minimal. Only the most recent HbA1c measurements were included in this study which may lead to an overestimation of the prevalence of diagnosed diabetes since the diagnosis needs to be confirmed with an additional HbA1c test. On the other hand some patients actually diagnosed with diabetes may be treated medically and thus have HbA1c levels below 6.5% (48 mmol/mol) leading to an underestimation of the prevalence of diabetes. Yet, the guidelines used in Greenland suggest targeting a HbA1c value below 7.0% (53 mmol/mol), and the number of patients with diabetes and a most recent HbA1c below 6.5% (48 mmol/mol) are expected to be of less significance. Another limitation in the estimation the prevalence of diagnosed diabetes and high risk pre-diabetes is that the patients included cannot be expected to be completely representative of Greenland’s population. Especially, the proportion of males examined is lower compared to females. Also, the patients tested with HbA1c may have increased risk factors for diabetes compared to the general population which would lead to an overestimation of the reported prevalence of diabetes. Thus, it can be expected that patients with overweight, obesity, hypertension, elevated blood lipids and other cardio-vascular symptoms would be offered an HbA1c test from the health care professionals more often than patients without these risk factors. This would tend to towards an overestimation of the prevalence of diabetes.

### Diagnostic activity

The proportion of patients (24.0%) tested with HbA1c in Greenland in the present study period is much higher than previously reported for the first 27 months after implementation of HbA1c as diagnostic tool in Greenland, indicating increased diagnostic activity. Thus, it was estimated that 13.6% of the population aged 35 years or above was tested during this study period [7]. Females were tested more often than males in the present study. This can be a consequence of gender specific differential use of health care service in Greenland. Thus, 90% of all females have been in contact annually with the health care system in Greenland compared to 76% of males [19]. In addition, all pregnant women in Greenland are routinely tested with HbA1c during the first trimester.

### Prevalence of diabetes

The prevalence of diagnosed diabetes at 4.3% among adults 20–79 years old is higher than the most recent (2014) reported prevalence of diagnosed diabetes in Greenland at 2.5%. In 2014 the prevalence of diagnosed diabetes among adults aged 20–79 years old were reported to be around 2.5% [16]. This may indicate that the prevalence of diagnosed diabetes is still on the increase in Greenland. On the other hand the prevalence is still lower than reported in the most recent population survey performed in Greenland during 2014. A prevalence of diabetes among adults aged 18 or above at 6.7% based on elevated glycated haemoglobin (HbA1c) tests performed on 537 participants was reported [2]. Of those, 60% were aware of their diagnosis indicating a prevalence of diagnosed diabetes, around 4% (60 of 6.7%), which is very similar to the prevalence at 4.3% among adults aged 20–79 years old reported in the present study. A prevalence of undiagnosed diabetes at around 40% of all diabetes cases is lower than the global average of approximately 45% of undiagnosed diabetes [2] However, despite high diagnostic activity in Greenland, undiagnosed diabetes remains an issue. Especially among males aged 40–70 years old, unawareness of diabetes may be present due to the lower proportion tested than from among females. The higher proportion of diagnosed diabetes and high risk pre-diabetes among females than males may partly be explained by the higher proportion of females tested partly because of the higher proportion of females with abdominal obesity among adult females compared to that of males in Greenland [20]. For both genders overweight and obesity have been increasing within the last two decades and may contribute to the increasing prevalence of diagnosed diabetes observed in Greenland [13, 21–23].

The prevalence of diagnosed diabetes in Greenland reported in this study seems to be quite similar or even a bit lower than in the surrounding countries. According to the International Diabetes Federation the prevalence of diabetes among 20–79 aged people is 7.6% in Iceland, 7.4% on the Faroe Islands, 9.9% in Denmark, 9.5% in Canada and 12.8% in the United States. Of those, 27–38% remain undiagnosed [24–28].

In conclusion, HbA1c is widely used in the health care system in Greenland indicating a high awareness of the occurence of diabetes in the population and a heightened awareness in the health care system, which may contribute to explaining the reported increase in the prevalence of diagnosed diabetes within the last few years. Still, awareness of undiagnosed diabetes remains an important issue, and additional strategies especially targeting males under 70 years old must be considered.
